# Interplays between gut microbiota and gene expression regulation by miRNAs

**DOI:** 10.3389/fcimb.2012.00137

**Published:** 2012-11-02

**Authors:** Andrea Masotti

**Affiliations:** Gene Expression – Microarrays Laboratory, Bambino Gesù Children's HospitalIRCCS, Rome, Italy

The gastrointestinal tract is one of the most colonized organs and harbors a large microbial population (10^14^ bacteria) that has co-evolved with us establishing a finely tuned symbiosis (Ley et al., 2008). As a result of their occurrence in complex mixtures, their combined genomes or metagenome, contain 150-fold more genes respect to humans, therefore providing us with novel functions (Qin et al., 2010). Increasing evidences show that the disruption of this symbiosis may lead to pathologies such as obesity or to an increased risk of developing inflammatory bowel disease (Ott et al., 2004). Since the intestine is constantly exposed to an almost limitless number of foreign antigens (i.e., food-derived materials, commensal microbes, pathogenic bacteria, viruses and parasites), it is of fundamental importance that an appropriate immune homeostasis in the gut mucosa is established and maintained. This requires a highly sophisticated immunological regulatory systems achieved by the cooperative interaction of intestinal epithelial cells (IECs) and mucosal cells of the immune response (Goto and Kiyono, 2011). The IECs comprise columnar epithelial cells, Paneth cells, endocrine cells and goblet cells (van der Flier and Clevers, 2009) and consist in the first physical barrier between the host and the external environment. Owing to many signals transmission from epithelial cells to the various innate and acquired types of mucosal cells of the immune response, these cells regulate each other resulting in intestinal immunological homeostasis.

To gain information about the composition of gut microbiota communities, their molecular interactions with the host and their impact on various host functional processes, several studies have been carried out on germ-free animals by coupling genomics and bioinformatics techniques (Hooper and Gordon, 2001; O'Hara and Shanahan, 2006). However, little is known about the molecular mechanisms of such modulations and the host post-transcriptional gene expression regulation by microRNAs.

MicroRNAs (miRNAs) are short (~22 nt) non-coding RNAs that control gene expression by base pairing with 3′-untraslated regions (3′UTRs) of their regulated transcripts. MiRNA biogenesis occurs through various steps in which are involved Drosha and Dicer, two main RNase III endonucleases. Precursor miRNAs (pre-miRNAs) are ~70 nucleotide-long RNA molecules with a characteristic hairpin structure. They originate in longer primary transcripts (pri-miRNAs) that are cleaved in animals by the Drosha endonuclease in the nucleus (Lee et al., 2003). Following the export of pre-miRNAs to the cytoplasm by Exportin-5, the loop region of the hairpin is removed by the Dicer endonuclease to produce a short, double-stranded RNA (dsRNA) (Cullen, 2004). Based on the thermodynamic stability of each end of this duplex (O'Toole et al., 2006), one of the strands is preferentially incorporated in the RNA-induced silencing complex (RISC), producing a biologically active mature miRNA (generally the -5p miR) (Bartel, 2004), while the inactive strand (the -3p miR) is degraded (Kim, 2005). The coupling of the active miRNA to the 3′UTR of its target gene, facilitates mRNA degradation or translation inhibition (Djuranovic et al., 2012). As a direct consequence, miRNAs regulate many biological processes and have critical roles in cell proliferation, differentiation and death (Shivdasani, 2006; Gomase and Parundekar, 2009).

**Figure 1:**
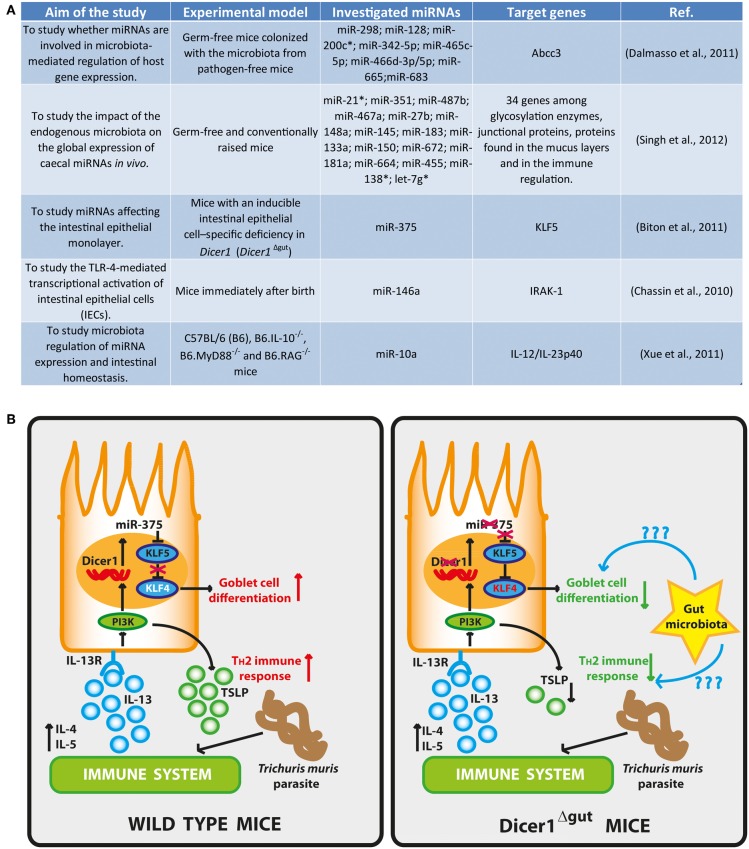
**(A)** Research projects for the study of the interplays between gut microbiota and miRNAs. **(B)** Intestinal epithelial differentiation and T_H_2 immune responses are regulated by miRNAs: in wild type mice, Dicer1 and miR-375 inhibit KLF5, a known antagonist of KLF4 that promotes the differentiation of goblet cells via KLF4. Helminth infection induces T_H_2 cytokines, especially IL-13, which leads to epithelial expression of miR-375 and goblet-cell maturation via PI3K. Moreover, miR-375 also induces TSLP to accelerate T_H_2 immune responses to parasite infections. In *Dicer1*^Δgut^ mice, depletion of Dicer1 or miR-375 results in fewer goblet cells and diminished T_H_2 responses. The gut microbiota can be involved in the induction and regulation of miRNA expression either for active or quiescent immunity. Likewise, other miRNAs can be involved in the generation of optimal protective immunity to various pathogens. This figure has been adapted from (Goto and Kiyono, 2011).

However, the role of miRNAs in microbiota host interactions is beginning to be investigated (Figure 1A) (Dalmasso et al., 2011; Kaser et al., 2011). Dalmasso et al. used germ-free mice colonized with the microbiota from pathogen-free mice to study whether miRNAs are involved in microbiota-mediated regulation of host gene expression (Dalmasso et al., 2011). Their miRNA expression analysis revealed that nine miRNAs were differentially expressed in the ileum and colon of colonized mice compared to germ-free mice. By overlapping the predicted targets of deregulated miRNAs with DNA microarray gene expression profiling, they found that the up-regulation of miR-665 induced a significant down-regulation of the ATP-binding cassette sub-family C member 3 (*Abcc3*) gene (a target of miR-665). Abcc3 belongs to the multidrug resistance-associated protein family, which mediates the metabolism of xenobiotics and endogenous toxins (Hooper et al., 2001). A similar study by Singh et al. emphasized the emerging interplay between endogenous microbiota and caecal miRNA signature (Singh et al., 2012). In fact, intestinal miRNAs have been proven experimentally to have roles in the regulation of neonatal nutrient metabolism (Liao and Lonnerdal, 2010), in the control of intestinal fluid and electrolyte transport (Sansom et al., 2010) and permeability (Zhou et al., 2010), affecting also intestinal epithelial cell differentiation (Dalmasso et al., 2010) and maturation (Zeng et al., 2009). By using germ-free and conventionally raised mice, the impact of the endogenous microbiota on the global expression of caecal miRNAs *in vivo* has been investigated, showing that the murine miRNA signature in the caecum is affected by the presence of the microbiota (Singh et al., 2012). Moreover, authors found that 34 putative miRNA target genes encode for proteins involved in the regulation of the intestinal barrier function (i.e., glycosylation enzymes, junctional proteins, proteins found in the mucus layers) and in the immune regulation (i.e., MHC I and II proteins). They found that the expression of miRNAs depends on the endogenous microbiota and that 16 unique miRNAs were deregulated between germ-free and conventional raised mice. By cross-matching the list of intestinal barrier genes predicted to be modulated by differentially expressed miRNAs, with genes already demonstrated to be deregulated in the jejunal mucosa of intestinal-specific Dicer knock-out mice (McKenna et al., 2010), the authors supported the hypothesis that gut commensals impact the intestinal barrier via miRNAs expression modulation. Therefore, the miRNAs modulation by gut microbiota may potentially affect the expression of a huge number of host genes, so far unexpected, especially in those diseases where the microbiota composition is altered toward less desirable species. In this context, the use of synthetic miRNAs could represent a potentially novel therapeutic perspective.

Another example of the role of miRNAs in affecting the intestinal epithelial monolayer, has been provided by Biton et al. by using mice with an inducible intestinal epithelial cell–specific deficiency in *Dicer1* (*Dicer1*^Δgut^) (Biton et al., 2011). They found that *Dicer1* deletion in the mice gut lead to goblet-cell depletion and that the regulation of goblet-cell differentiation is dependent on the expression of miR-375. In fact, the expression of this miRNA is able to inhibit the translation of KLF5, an antagonist of the goblet cell–differentiation factor KLF4, supporting the differentiation of goblet cells. Moreover, they observed a lower expression of IL-4, IL-5, and IL-13 in *Dicer1*^Δgut^ mice and an enhanced susceptibility to helminth parasite *Trichuris muris* infection (Figure 1B). IL-13, presumably supplied by T_H_2 cells, induces miR-375 in IECs *in vitro* and a down-stream production of the T_H_2-facilitating epithelial cytokine TSLP, indicating an appropriately balanced T_H_2 feed-forward loop regulated by miR-375. On the basis of their results, the authors suggested that miR-375 directs the differentiation of goblet cells and the promotion of antiparasitic T_H_2 immune responses. As miR-375 expression is very high in the human intestine (Wu et al., 2010), mucosal expression of this particular miRNA might also be important in the regulation of intestinal homeostasis and protection against parasite infection in humans (Goto and Kiyono, 2011). Further investigation should allow answering to many open questions still remaining, such as whether there are other miRNAs involved in this process or whether there are other miR-375 targets relevant to the differentiation of goblet cells or in the maintenance of gut immunological homeostasis. It is quite easily conceivable that in a near future we will assist to the development of innovative mucosal miRNA–targeted treatments and to the diagnosis of pathogenic mucosal conditions such as allergy, inflammatory bowel diseases and colon cancer, as well as infection by bacteria, viruses and parasites by employing specific miRNA-designed tests.

One of the most recently emerging and appealing concept is the role of toll-like receptors (TLRs) as potential mediators between gut microbiota and miRNAs/mRNAs modulation in humans. In fact, it has been recognized clearly that host gene expression is regulated by gut microbiota along the length of the gut and that microorganisms recognition is mediated by TLRs through the adaptor molecule MyD88 (Dalmasso et al., 2011; Larsson et al., 2012). Therefore TLRs, localized at the interface between the microbiota and the molecular machinery of host cells, may be key players in these relationships. In a recent study, Chassin et al. found that the TLR-4-mediated transcriptional activation of IECs observed in mice immediately after birth, was induced by oral ingestion of environmental endotoxin and induced a post-transcriptional down-regulation of epithelial IRAK1 protein expression, protecting further from bacteria-induced epithelial damages (Chassin et al., 2010). According to evidences showing that IRAK-1 expression is regulated by miR-146a (Taganov et al., 2006), miR-146a levels declined only in concomitance to the increase of IRAK-1 protein level, whereas miR-146a silencing induced IRAK1 protein expression. Moreover, authors demonstrated that the oral treatment with anti-miRNAs is a viable option to down-regulate the expression of miR-146a in intestinal epithelial cells.

Another study focusing on the microbiota regulation of miRNAs expression and on the maintenance of intestinal homeostasis, has been reported by Xue et al. who reported a connection between the expression of miR-10a and of its target IL-12/IL-23p40, a key molecule for innate immune responses to commensal bacteria (Xue et al., 2011). They also found that commensal bacteria down-regulated dendritic cell miR-10a expression via TLR–TLR ligand interactions through a MyD88-dependent pathway and that mice with colitis expressed higher levels of IL-12/IL-23p40 and lower level of gut miR-10a, compared to control mice, opening new perspectives for the study of miRNAs regulation in intestinal diseases.

In the field of RNA silencing, very close to that of miRNAs for their common mechanism of action, a novel approach exploiting engineered bacteria has been reported few years ago (Xiang et al., 2006). This approach holds great promise for functional genomics in mammalian systems and for other *in vivo* applications, since it demonstrates that the trans-kingdom RNA interference (RNAi) process is feasible both *in vitro* and *in vivo*. Authors employed *E. coli* engineered to produce short hairpin RNAs, by the use of a plasmid that they termed TRIP (trans-kingdom RNAi plasmid). This vector contains *Inv* and *HlyA* encoding for invasin, and listeriolysin O, respectively, enabling the entry into β1-integrin-positive mammalian cells and the release of genetic materials from internalized vesicles. By co-culturing human colon cancer cells (SW480) *in vitro* with the engineered *E. coli*, a significant down-regulation of a specific target gene has been observed, demonstrating the effectiveness of the trans-kingdom RNAi mechanism *in vitro*. By oral or intravenous administration of the engineered *E. coli*, the authors demonstrated also an efficient gene silencing in the intestinal epithelium and in human colon cancer xenografts in mice, suggesting a clinically feasible approach to the *in vivo* application of RNAi technology. Most interestingly, this trans-kingdom RNAi approach not only can be exploited clinically to silence genes in the colonic mucosa and in other organs colonized by bacteria (i.e., oral cavity, urinary bladder, and female genital tract), but also suggest the speculative but intriguing possibility that such RNAi mechanism may occur also in natural interactions such as infections, commensal interaction and symbiosis.

In conclusion, I described two works (Dalmasso et al., 2011; Singh et al., 2012) dealing with a differential expression of miRNAs in different areas of the intestinal tract as a function of microbiota composition. In these cases, intestinal microbiota are the “actors.” Conversely, we should also think to miRNAs as “actors” when, under proper conditions, influence the regulation of goblet-cell differentiation (Biton et al., 2011). Therefore, an interconnected cycle could be envisaged where miRNAs and gut microbiota are the two main partners. Two interesting approaches (use of engineered vectors and the oral delivery of anti-miRNAs) (Chassin et al., 2010; Xue et al., 2011) have emerged as interesting possibilities to study experimentally the interplays between miRNA and gut microbiota, and their mutual role in influencing host immune system and related processes. Therefore, although still a few, the studies reported so far emphasize that we cannot ignore that “our other genome” is intimately linked to “our natural miRNome.”
